# “It’s That They Treated Me Like an Object”: A Qualitative Study on the Participation of People Diagnosed with Psychotic Disorders in Their Health Care

**DOI:** 10.3390/ijerph20054614

**Published:** 2023-03-05

**Authors:** Amelia Villena-Jimena, José Miguel Morales-Asencio, Casta Quemada, María M. Hurtado

**Affiliations:** 1 Mental Health Unit, Regional University Hospital, 29009 Málaga, Spain; 2 Faculty of Heath Sciences, University of Málaga, 29071 Málaga, Spain; 3 Instituto de Investigación Biomédica de Málaga y Plataforma en Nanomedicina-IBIMA Plataforma BIONAND, 29590 Málaga, Spain

**Keywords:** psychotic disorder, schizophrenia, shared decision making, patient participation, patient-centred care

## Abstract

The mental health recovery model is based on shared decision making, in which patients’ preferences and perceptions of the care received are taken into account. However, persons with psychosis usually have very few opportunities to participate in this process. The present study explores the experiences and perceptions of a group of patients with psychosis—in some cases longstanding, in others more recently diagnosed—concerning their participation in the decisions taken about the approach to their condition and about the attention received from healthcare professionals and services. For this purpose, we performed a qualitative analysis of the outcomes derived from five focus groups and six in-depth interviews (36 participants). Two major themes, with five sub-themes, were identified: shared decision-making (drug-centred approach, negotiation process, and lack of information) and the care environment and styles of clinical practice as determinants (aggressive versus person-centred environments, and styles of professional practice). The main conclusions drawn are that users want to participate more in decision making, they want to be offered a range of psychosocial options from the outset and that their treatment should be based on accessibility, humanity and respect. These findings are in line with the guidelines for clinical practice and should be taken into account in the design of care programmes and the organisation of services for persons with psychosis.

## 1. Introduction

In the 1990s, a new paradigm for mental health, the Recovery Model [1,2], was proposed. This had a significant impact on the approach taken to patients with psychosis, encouraging the active involvement of users in coping with their disorder, and enabling their own preferences, values, motivations and goals to be included in the treatment plan. With this approach, therefore, patients become the protagonists of their recovery process and their preferences and suggestions regarding the health services offered must be heard and taken into account.

The concept of shared decision making (SDM) is of particular importance in the Recovery Model [3,4]. Furthermore, it is supported by the WHO [5], is included in policies to promote the co-production of health services [6,7,8,9] and is consistent with several guidelines published in this respect [10,11,12].

SDM is based on four assumptions: (1) at least two people are involved, one of whom is an expert in a given subject area, and another in his or her own life; (2) available information is shared; (3) the treatment decided upon should be negotiated between these persons; and (4) the treatment has a time limit [13]. Among other benefits, the adoption of SDM has been associated with alleviated symptoms, improved self-esteem, increased satisfaction with medical care, better treatment adherence and decreased rates of hospitalisation [14]. However, much remains to be done in this regard [15]. Although studies have reported that most patients prefer this treatment model [15,16,17,18,19,20,21,22], many who are being treated for a mental disorder believe they do not participate enough in their healthcare decisions [23,24]. The main guidelines for the treatment of psychoses propose that initial interventions should be pharmacological, psychological, related to physical health care, and be educational and/or employment-related [25,26]. In clinical practice, however, an initial drug-based approach is commonly adopted [27,28]. Furthermore, most research in this field has focused on pharmacological treatment [29], on identifying facilitators and barriers with respect to patients, professionals and the health system [15,29,30,31,32] and/or on specific strategies to facilitate decision-making [33]. Regarding the negotiation process between patients and care providers, however, much less is known.

Mental health patients have expressed their wishes for healthcare professionals and services to respect their autonomy and to propose treatment approaches in line with their needs [34]. However, some of those with severe mental disorders have complained that they do not receive this sort of person-centred health care [35]. Awareness of, and willingness to act upon, patients’ preferences could both enhance the interventions made and foster perceptions of empowerment [36,37]. In addition, the current trend towards more person-centred care for patients with psychotic disorders makes it necessary to continually review these aspects of treatment.

In view of these considerations, the aim of the present study is to examine the experiences and perceptions of a group of patients diagnosed with a psychotic disorder, with particular regard to the decision-making process adopted and to the relationships between these patients and the attending health professionals and services. We also consider whether persons who have been recently diagnosed with psychosis and are participating in intensive intervention programmes have different kinds of experiences and perceptions from those reported by persons whose condition is of longer standing and who have not participated in such programmes.

## 2. Method

### 2.1. Study Context and Sample

This qualitative study was carried out at the Mental Health Service of the Regional University Hospital of Malaga (Spain), which serves a population of 165,000 inhabitants. Care services for persons with psychosis include acute hospitalisation and specialised outpatient care in the community.

The study participants were selected by purposive sampling in accordance with the following criteria: having been diagnosed with a psychotic disorder (in the ICD-11 terminology, F20 Schizophrenia; F21 Schizotypal disorder; F22 Persistent delusional disorders, F23 Acute and transient psychotic disorder or F25 Schizoaffective disorder) and currently being monitored by the above Mental Health Service. Participants who presented significant psychotic symptoms and whose onset was for less than one year were also admitted, even if they did not have an established diagnosis but rather a provisional one. People who were currently experiencing active symptoms that might influence their participation were excluded. All those who fulfilled the inclusion criteria were telephoned to explain the objectives of the study and to request their participation. Those who were subsequently interviewed were again informed about the study goals, assured that all data would be treated in strict confidence and asked to sign the informed consent form. The sample size was determined in accordance with the principle of data saturation during data collection and analysis.

### 2.2. Analyses

The study data were compiled from two sources: focus group semi-structured interviews and in-depth interviews. In the focus groups, the time elapsed since diagnosis was the only segregation variable used. The topics proposed to the participants were determined by the researchers on the basis of a prior literature review and by expert consensus (see Supplementary Materials). The following topics were discussed: information about the disorder, treatments offered and ways of participation in decisions and proposals concerning their mental health. The interviews lasted 90–120 min, and were carried out by a neutral interviewer, who was highly experienced in conducting qualitative interviews. Moreover, an observer took notes on the situation of each participant and the non-verbal aspects that might aid in understanding of the interactions among participants. The interviews took place at a health centre other than the one where usual care was provided.

All interviews were recorded, and the audiotapes were transcribed verbatim, after which a content analysis was performed according to the principles suggested by Taylor et al. (2015) [38]. The transcripts were read to identify the main themes addressed and were subsequently coded by a member of the research team. These codes were then triangulated, with reviews by two other members of the research team. Any differences in the codes proposed were discussed and resolved among the researchers. The codes were grouped into categories and subcategories and analysed taking into account potential researcher bias. All analyses were performed using ATLAS.ti (version 7, Berlin, Germany) software for qualitative data analysis.

The criteria of credibility, transferability, consistency and confirmability, as identified by Guba and Lincoln in this respect [39], were considered. To ensure the credibility of the analysis process, all codes and categories were triangulated. Transferability was strengthened by ensuring the completeness of data collection in each group, across multiple potential situations, scenarios and experiences with psychotic disorders. The criteria of data consistency and reproducibility were achieved by a detailed and documented description of the analysis process and the context in which data collection took place. With respect to the assurance of confirmability and reflexivity, the researchers conducted an analysis of their own preconceptions and expectations, before the start of the study, regarding the study results, to consider the extent to which these might subsequently influence the study procedures. Additionally, the moderator/interviewer for the qualitative interviews was neutral (not a member of the research team) and was highly experienced in this field.

### 2.3. Ethical Aspects

All participants provided written informed consent to participate in the study. The Malaga Ethics and Research Committee approved this study. The principles of good practice and the provisions of the Declaration of Helsinki and its subsequent revisions were upheld throughout the study. The data were treated confidentially and pseudonymised for statistical analysis.

## 3. Results

Two broad categories were identified: shared decision making (SDM) and aspects of the treatment relationship and of the environment. In this sample, we have not observed significant differences between the experience between men and women with respect to their participation in the health care process; rather, their discourses tend to converge on the same themes. The study was conducted with 36 participants, who took part in five focus groups and provided six in-depth interviews. The characteristics of the participants are detailed in Table 1.

### 3.1. Shared Decision Making

Three subcategories of SDM were identified: drug-centred approach, the negotiation process and the lack of information.

#### 3.1.1. Drug-Centred Approach

Pharmacological treatment is usually prescribed during the first consultation with the psychiatrist, the mental health professional who normally provides the first care attention. In some cases, if the treatment is initially rejected, the prescription is postponed and reconsidered in subsequent visits:

*What I remember, with the first psychiatrists, it was medication; I’d arrive there for the consultation and, well, I’d tell them my story.*

(Male, 33)

*At first, I didn’t want the medication. In the first consultation, I said no. And in the second one he said again that he had to put me on medication. In the end, he convinced me and they gave me some pills.*

(Female, 29)

Usually, patients accept this first prescription. But they are often reluctant to continue, because of worries about possible side effects, or they are not fully aware of their own health problem, or even because they perceive an initial improvement.

*Well, I don’t know if it’s good or bad for me, but I take it. I know I have to take it… it’s just a routine.*

(Male, 48)

Patients frequently express a preference for another type of therapy, to complement the use of drugs. Users with longer experience of this type of disorder, especially, tend to prefer alternatives to the pharmacological approach:

*The last time I had a problem, they said I needed “An antidepressant”. But I didn’t take an antidepressant… I’ll do something else. I’ve signed up for swimming, to do yoga, to go for walks if I feel like it… I do things that I like.*

(Female, 42)

Users prefer it when healthcare professionals make suggestions, rather than imposing their views.

*They told me “You’re taking this, it seems to be going well, but it could be even better, do you want to try Leponex and see how you do with that?”. And I said yes because I really wasn’t doing that well, anyway…*

(Female, 49)

Regarding the offer of treatment options as an alternative or complement to pharmacological methods, patients differ according to when the first episode of psychosis took place. Offers of options and referral to other resources are more commonly made from the outset to patients whose disorder appeared relatively recently. The experience of users with a longer history of psychosis tends to be restricted to (sporadic) psychiatric treatment and (more regular) nursing follow-up. The contrast in these types of attention has been remarked upon, as has the perception of closer attention and accompaniment from the nursing team.

*As far as I’m concerned, more than the psychiatrist, really, it’s the nurse who looks out for me…, the psychiatrist too, but the truth is, before, the psychiatrists didn’t talk to you, they just looked at you, prescribed the medication and goodbye… they’re always in a hurry.*

(Female, 42)

Almost all patients have participated at some time in group therapy sessions, mutual aid groups, alternative therapies recommended by healthcare professionals or family therapy, or have been referred to activities organised by the third sector. Patients with a more recent onset of the disorder usually have earlier access to these resources. In general, they are highly satisfied with this type of intervention.

*You have a better understanding of what’s happening to you and why it’s happening to you. And you analyse this, you carry out an introspective analysis, with a different approach. These are very positive aspects, and above all, later you form friendships within the group. In principle, everything about it is positive.*

(Male, 48)

*I used to spend a lot of time in my room, at home and I… I didn’t want to go out. That all changed when they said “Come on, it’s going to be like in football, it’s something good, it makes you feel good, you socialise…”.*

(Male, 24)

#### 3.1.2. Negotiation

Many patients report that in initial consultations they are not invited to choose from pharmacological alternatives. In addition, most have confidence in professional judgment, which means they are unlikely to question the suitability of the treatment initially offered.

*The problem is that… you didn’t have a point of reference. It’s just that… you arrived, you found yourself in this situation and they set out the options. “You’re the specialists, you know what you’re doing”.*

(Male, 24)

A significant number of patients reported this non-participation in decision-making regarding their treatment, arguing that the professional is best qualified to determine the treatment approach.

*It’s the psychiatrist who decides on the change from one medication to another… Anyway, I think it’s his duty…*

(Male, 33)

On other occasions, it is the psychopathological state itself that impedes participation, for example, if the patient is in a state of catatonia and can barely express himself, or simply does not understand what is happening to him.

*Because if you know what’s happening to you, you can tell someone and they can help you, but if you can’t express what is happening to you, or what’s the matter, who’s going to help?*

(Female, 42)

However, a significant number of patients do wish to negotiate their treatment, especially when their side effects become less tolerable.

*When I used to say, maybe, that I was really stressed, sleeping very badly, that sort of thing, she’d say “Take a Lorazepam”; and I’d tell her “I can’t take a whole one because then I’d sleep for 17 h”. So, she’d say “Take half a one, then”. “OK, I’ll take half a one”, I said, that’s how it went. Between her and me, we talked it over and she advised me, more or less, how it would suit me. So, I felt good in that respect.*

(Female, 24)

Sometimes, negotiation does not bring about the desired results, and this might lead to the patient abandoning the medication.

*I have to argue quite a lot. They changed my treatment thanks to the nurse.*

(Male, 50)

*…they put me on RISPERDAL too, but my period stopped and I didn’t want it. But they said I had to take it anyway. But that didn’t seem logical to me.*

(Female, 52)

Many patients whose first psychotic episode occurred more than ten years ago complain that, initially, only the pharmacological approach to treatment was offered. They would have liked to be offered the alternative of therapy from the outset rather than at a later stage.

*What I do think is that maybe there should be a little more therapy, a little more conversation.*

(Male, 33)

Recently, programmes have been offered in response to the first psychotic episodes, enabling psychotherapeutic intervention from an early stage and facilitating better follow-up attention by a multi-professional team. This careful monitoring means that broader-based attention can be provided, in accordance with individual needs. The following testimony describes the usefulness of the follow-up provided by a clinical psychologist to a patient who continued to use cannabis despite realising that this might hinder her recovery.

*So, she made appointment after appointment for me, and I used to say “Well, if I’m fine, why does she keep giving me another appointment, every week, or every other week?”. I think it did me good, too, because every time I left, I’d think “No, the next time I come, I won’t be smoking anymore”, and I had that thought in my mind. In the end, I didn’t even realise …. But, that obligation of “Come on. You’ve got to go and see her. You’ve got to do it right”… Although it’s true that later I’d say “Damn, I’ve got to go tomorrow. It’s been two weeks and it’s the same again. Well, come on, by next time…”. For me, personally, it’s helped a lot.*

(Female, 24)

Finally, despite being explicitly asked, none of the participants knew what advance directives were.

#### 3.1.3. Absence of Information

It seems to be fairly common for patients not to receive information about their diagnosis from the outset, albeit provisional and subject to change. In some cases, the patient discovers the diagnosis after requesting a report needed for an unrelated procedure or from reading the leaflet accompanying the antipsychotic medication prescribed.

*I picked up the psychiatrists’ papers to find out exactly what I had… they were related to some forms in the court proceedings (…) and that was when I found about the disease I had.*

(Male, 24)

*I think they should explain a little more… “You have a disease, it has these symptoms… this occurs very frequently”… Before this, I even read the package insert for a medication for schizophrenia, and it described exactly what was happening to me.*

(Male, 51)

Some people even went so far as to search the internet for information because they felt that they did not know enough about what was happening to them, but this experience did not turn out well.

*I took a sheet of paper, some pencils, pens… let’s see, this website says, ufff… Well, let’s see this one, ufff. On the other hand, ufff, this doesn’t look very good either,… well, that’s it, shut down the computer and forget about the world.*

(Female, 24)

Some patients have no special interest in finding out their diagnosis in this initial phase. Indeed, many emphasise they would rather have this information later, when their clinical situation has stabilised, or want the diagnosis reconsidered later, with greater certainty.

*At that moment (on being discharged from the first hospital admission), I was still very dazed, and at that moment it really wouldn’t have helped me if they had told me the diagnosis. It would have been worse, because what I wanted was to get out of there and rest, I mean, to be calm, you know?*

(Female, 24)

*You’ve got to be careful, words are powerful… The biggest shock I’ve ever had was when I went to the psychiatrist, and I was diagnosed without any physical tests, without an fMRI or imaging tests… “You’ve got this”.*

(Male, 24)

Generally, in these patients’ experiences the information on their diagnosis is given little by little during the care process, and this information is provided by all the healthcare professionals involved.

*In my case, they told me from the first moment when the symptoms appeared. I told the doctor “Look, the police are chasing me”. He said, “Look, J., the police can’t be chasing you, because a patrol car is not going to be chasing you exclusively.” They also explained to me about the cars, which were delusional signs. The nurses told me all about the disease, what it was like…*

(Male, 50)

Many patients remarked that they talked with the professional regarding any doubts they had about the treatment and the side effects experienced.

*A bit doubtfully, I asked her (the psychiatrist), “Is this medication for schizophrenia?”, and she said, “No, but it can also be used to treat that”, and then she explained it a little more, and so I got used to the idea.*

(Male, 24)

In several of the interviews, the patients expressed concern about the duration of their treatment, believing this issue should have been addressed in the consultations.

*What about the medication? Nothing, you just have to take it. That’s all. Why? Because right now you need it. End of discussion. No end date. Indefinite. Indefinite, it might be three weeks, it might be a month, it might be six… (…) We’ll see how you come along.*

(Female, 24)

Patients being treated in some current intervention programmes expressed their awareness of opportunities during meetings for raising concerns and fears regarding their condition, possible relapse or withdrawal from treatment.

*(The psychiatrist) told me “You don’t choose the illness, it chooses you and your number came up” and I said, “Jeez!”. And the thing is, he’s right. It’s up to me to resolve this, that’s all, get over it. I didn’t choose it. So, I won’t get stressed about it.*

(Female, 24)

*A few months ago, when the anniversary of my admission was coming up, I told him (the psychiatrist), “I’m scared.” I was afraid it was going to happen again.*

(Female, 24)

### 3.2. The Care Environment and Styles of Clinical Practice as Determinants

The category of “Care environment and styles of clinical practice as determinants” contains two subcategories: coercive vs. person-centred environments; and styles of professional practice.

#### 3.2.1. Coercive vs. Person-Centred Environments

The experiences described in the patients’ interviews differ according to whether attention was received as part of outpatient or of hospital care. In the former case, respondents highlighted the ease of accessibility, the adaptation of care to individual circumstances, the priority granted at all times to the patient’s needs and the provision of a space in which worries and fears could be addressed. By contrast, the hospital environment is seen to be more rigid and the care provided, more hostile; patients refer to the use of coercive measures, the experience of objectification and the scant occupational options provided. Some, however, with longer experience as users of the health system, remarked that the application of coercive measures had changed, over time, during their admissions.

*In the past, quite some time ago, when I was first admitted to a hospitalisation unit, the first thing they did was tie you to the bed, whether you were being aggressive or not. (…) Now, that has changed.*

(Male, 42)

However, although there have been changes in this regard, the experience continues to be one of rigidity in the rules applied, and the patients are still treated in a paternalistic way.

*They treated me like an object. They didn’t explain anything. They didn’t even say, “Look, we’re going to do this or that”. Not a single word of reassurance. “Look, don’t worry, nothing bad will happen”… Before that, I’d never been treated as if I couldn’t understand things*

(Female, 29)

In addition, the respondents made specific proposals for improvements in aspects such as shared rooms, occupational activities in the hospital unit and ambulance transfers.

*Sharing a room with other people (..) Being locked up… You’re not well, but the person next to you isn’t well either. You know how you are going to react, but you don’t know how that other person is going to react (…). So, you might freak out. Like I said, “Hell, they’ve closed the door on us here”*

(Female, 24)

*I think that when you’re hospitalised you have too much time to think, you and everyone else around you. I think it would be interesting to do activities (…). Like yoga, playing ball, football…*

(Female, 24)

Conditions during admission such as those described above, together with the patient’s state of anguish, suspicion and delusional tendency, typical of the acute phase, may contribute to the admission experience being termed traumatic by some.

*They came from the ambulance, and I thought they wanted to catch me. I ran away. They grabbed me anyhow. That was very traumatic, because one of the ambulance men got me, threw me to the ground, held me there, squashing me… I couldn’t do anything. Not being able to defend myself… Really, I had a terrible time.*

(Female, 29)

#### 3.2.2. Styles of Professional Practice

Some respondents expressed their wish for health professionals to display empathy, accessibility, humanity and trust.

*When you’re very unwell, knowing that you can call your doctor and talk to him, that’s priceless, really*

(Male, 33)

*But later, when I met this team, people who listened to me, who gave me my half hour every month to talk about my anxieties, my problems or my achievements, that’s when you feel that people are on your side*

(Female, 42)

Other areas that are highly valued by the patients consulted include personal validation, the return of positive aspects in life and receiving optimistic messages from healthcare professionals.

*My psychologist has always told me that I’m very strong, that I’m a person who has obtained a university degree, that I’ve had a job… And maybe I didn’t realise all that, but now I do and I say “I’ve been very ill, but I’ve done all this”. And all those comments did me good, they gave me a little reminder that I am worth something*

(Female, 24)

*What really helped me was when they said, “Don’t worry, this will take time, but you’re going to be fine”. That phrase was constantly repeated and it helped me a lot*

(Female, 29)

## 4. Discussion

In this study, we analyse the experiences and perceptions of a group of patients diagnosed with a psychotic disorder, considering the extent to which they participate in the decisions taken regarding their treatment, and their relationship with the mental health professionals and services involved. Our findings show that these patients do not always take part in shared decision making, although it seems to be their preferred option in almost all cases. A major difference between patients whose condition has been diagnosed more recently and those with a long-standing psychotic disorder is that the former are usually offered a wider range of treatment resources in the early phases of care. Our study also highlights the importance of certain aspects of healthcare personnel and the therapeutic environment that favour the patient’s satisfaction with the care received and which promote feelings of trust, thus facilitating adherence to treatment. Finally, some proposals are made for improvement in this regard.

Regarding treatment options, pharmacological treatment and psychological intervention should be offered from the outset, together with employment assistance or training programmes as and when the patient wishes [25,26]. According to the patients consulted in this study, pharmacological treatment is usually offered from the beginning of the care process, but only those who have been recently diagnosed are also offered a psychological intervention from the very start. The introduction of early intervention programmes for patients with psychosis could explain why psychosocial intervention is now offered at an initial stage [40]. However, all patients are offered this type of intervention sooner or later, as well as referral to employment or training programmes, enabling them to benefit from the proven efficacy of psychological and psychosocial interventions, concerning both the symptoms of their condition and the functions of everyday life [41,42].

Regarding therapist–patient negotiation, the initial decisions on treatment are usually taken by the professional, without debate, and negotiation only arises if unpleasant and/or annoying side effects occur. This absence of negotiation, in the initial stages of treatment, may be due to the psychopathological state itself or to the absence of a culture or referents for negotiation on health-related decisions [30]. The alteration of the cognitive functions detected in these patients can also be an important drawback during the negotiation process [43,44]. Frequent contact with healthcare professionals is believed to facilitate this negotiation; it also enables therapists to respect the patient’s rhythm and fosters the perception of self-efficacy. In contrast, the lack of time and reduced accessibility are identified as systemic barriers to shared decision making [14,45].

Patients commonly receive little information about their diagnosis during the initial stages of consultation, and only become better informed as the care process advances. However, this is logical since a firm diagnosis cannot be made without observing the evolution of the symptoms [26]. In fact, one patient remarked on the shock experienced when he received an early diagnosis of schizophrenia. This finding corroborates previous qualitative studies in this respect [46,47]. However, a risk that could arise from the perceived absence of information is that patients might search for information from unreliable sources, such as the internet [48]. Indeed, up to 61% of patients with schizophrenia report having searched the internet for information about this disorder and its treatment [49]. It seems, therefore, that a different approach is needed, in the form of a model by which information can usefully be provided at an early stage: on the one hand, avoiding the over-hasty classification of a health problem, but at the same time, alleviating the uncertainty which can spur patients to seek unverified information elsewhere.

Many patients want to know, specifically, how long their treatment will last. In this respect, however, clinical practice guidelines do not refer to a precise duration, but merely state that the pharmacological treatment prescribed in the first psychotic episode should be maintained for one to two years [25,26].

Finally, there is a significant difference between patients’ perceptions of outpatient care and of mental health hospitalisation services, with the latter being viewed as more hostile, more rigid and less inclined to include patients in the decision-making process. In this respect, previous studies of hospitalisation have reported that both patients and staff have complained of bureaucratic rigidity, restrictive care protocols and (in many cases) a failure to obtain informed consent [50]. Some studies even characterise hospitalisation as a traumatic experience [50,51,52,53]. In our own study group, some of the patients made a similar assessment, and proposed measures to ease the discomfort often felt during hospitalisation and reduce feelings of fear, uncertainty and objectification. These proposals include explaining what is being done, giving patients individual rooms and increasing the quantity and quality of scheduled activities during hospital admission. Nevertheless, patients who had been receiving treatment for a longer period did acknowledge improvements in the care provided and the decreased use of coercive measures. To achieve the latter result, previous studies have recommended greater involvement by patients, shared decision making [54,55] and wide-ranging strategies to re-direct inpatient psychiatric services towards patient-centred care [56]. Moreover, in the case of psychotic disorders and perhaps all serious mental disorders that tend to have a chronic course, SDM can be understood as an iterative model, wherein upon discharge patients are asked to reflect on the admission and provide feedback on how they viewed the decision-making process, particularly if the patient was coerced into receiving a pharmaceutical intervention. This can take the form of an advanced directive, de-facto enclosing the SDM process in a legal framework, or informally through the creation of a Ulysses clause whereby the patient may consent in advance to future coercion for the sake of his/her best-interest. Users of mental health services are highly interested in PADs and regard them as tools to improve their involvement in care, preferring legally binding PADs that can be revoked only when users are competent to consent [57].

The results obtained in the present study also illustrate patients’ preferences for the therapeutic relationship: they wish to be treated in a warm, human, empathetic way; they want the treatment experience to validate their own reality; and they need to receive positive feedback on the evolution of their condition. In short, a good relationship with healthcare personnel facilitates the patient’s involvement, recovery and satisfaction [58,59].

Research with users with common mental disorders also shows the same preference for shared decision making that is not always satisfied [60]. However, a previous study carried out by the same research group with patients with generalized anxiety disorder in the same healthcare context revealed that user involvement was low and although pharmacological treatment was always the first option offered, almost all received psychological intervention in the first contacts [61]. Given these differences, it is worth wondering if the results obtained with psychotic patients in this study represent a specific diagnostic problem or if they tend to appear when patients with chronic mental disorder enter treatment.

The present findings should be interpreted in the light of certain limitations. Firstly, there may have been some self-selection bias, in that the analysis only considered the patients who agreed to participate in the focus group. Those who did not wish to take part or who ultimately did not attend might have had different healthcare experiences from those we describe. This decision could be influenced by the severity of their disorder when invited to participate, or by dissatisfaction with the healthcare received. Secondly, all the participants were treated in the same health district, which might compromise the generality of the results obtained. Third, most of the participants have had their psychotic illness for several years. People with a shorter evolution time are likely to have a different experience, as the data suggest.

## 5. Conclusions

This study, in itself, is an example of patients’ participation in their own healthcare. On the one hand, it highlights the current state of affairs regarding shared decision making, and on the other, it identifies the most and the least appreciated aspects of patients’ relationships with healthcare professionals and the treatment setting. According to the patients consulted, they want to be more involved in the decisions taken about their treatment, and believe that a range of interventions (pharmacological, psychological, physical healthcare, training or employment-related) should be offered from the outset, in line with clinical practice guidelines.

Ensuring these requests are met would foster a closer, more effective relationship and reduce the probability of hospitalisation becoming an aversive experience.

## Figures and Tables

**Table 1:** Characteristics of the participants.

I.D.	Gender	Age	Diagnosis	Age of Onset
G1P1	Male	40	Schizophrenia and recurrent depressive disorder	22
G1P2	Female	42	Schizophrenia	18
G1P3	Female	49	Schizoaffective disorder	20
G1P4	Female	37	Schizophrenia	Unknown
G1P5	Male	38	Schizophrenia and schizotypal disorder	17
G1P6	Male	37	Schizophrenia	20
G1P7	Male	33	Schizophrenia	18
G1P8	Female	43	Schizophrenia	20
G1P9	Male	31	Psychotic disorder	24
G1P10	Female	50	Schizophrenia	29
G1P11	Male	43	Schizophrenia and schizotypal disorder	22
G2P1	Female	63	Persistent delusional disorder and recurrent depressive disorder	39
G2P2	Female	54	Schizophrenia	28
G2P3	Female	29	Schizoaffective disorder	24
G2P4	Male	48	Schizophrenia	31
G2P5	Female	45	Schizophrenia	33
G2P6	Female	55	Schizophrenia	33
G2P7	Female	59	Schizophrenia and recurrent depressive disorder	34
G2P8	Male	68	Schizophrenia	30
G3P1	Male	50	Schizophrenia	24
G3P2	Male	41	Schizophrenia	14
G3P3	Female	50	Schizophrenia	32
G3P4	Male	51	Schizophrenia	41
G3P5	Male	42	Schizophrenia	Unknown
G3P6	Male	24	Schizoaffective disorder	22
G3P7	Female	59	Schizoaffective disorder	30
G3P8	Female	49	Schizophrenia and recurrent depressive disorder	27
G3P9	Male	41	Schizophrenia	27
G3P10	Female	51	Schizophrenia	30
EP1	Male	33	Schizophrenia	17
G4P1	Male	24	Severe depressive episode with psychotic symptoms *	23
G4P2	Female	24	Acute transient psychotic disorder *	20
G4P3	Male	21	Psychotic disorder *	20
EP2	Female	24	Psychotic disorder *	18
G5P1	Female	24	Acute schizophrenia-like psychotic disorder *	24
G5P2	Male	25	Psychotic disorder *	18

* Diagnosis proposed at the first months of health care and should be considered provisional.

## Data Availability

The dataset supporting the conclusions of this article is available in the *Institutional Repository of the University of Málaga* (RIUMA), in https://hdl.handle.net/10630/26081.

## Supplementary Materials

The following supporting information can be downloaded at: https://www.mdpi.com/article/10.3390/ijerph20054614/s1, Interview Script.

## References

[1] Davidson L., Roe D. (2007). Recovery from versus Recovery in Serious Mental Illness: One Strategy for Lessening Confusion Plaguing Recovery. J. Ment. Health.

[2] Shepherd G., Boardman J., Slade M. (2008). Making Recovery a Reality|Centre for Mental Health.

[3] Duncan E., Best C., Hagen S. (2010). Shared Decision Making Interventions for People with Mental Health Conditions. Cochrane Database Syst. Rev..

[4] Slade M. (2017). Implementing Shared Decision Making in Routine Mental Health Care: Forum—Shared Decision Making in Mental Health Care. World Psychiatry.

[5] World Health Organization (2013). Exploring Patient Participation in Reducing Health-Care-Related Safety Risks.

[6] Batalden M., Batalden P., Margolis P., Seid M., Armstrong G., Opipari-Arrigan L., Hartung H. (2016). Coproduction of Healthcare Service. BMJ Qual. Saf..

[7] People and Communities Board (2016). Six Principles for Engaging People and Communities: Putting into Practice.

[8] People and Communities Board (2016). Six Principles for Engaging People and Communities: Definitions, Evaluation and Measurement.

[9] Elwyn G., Durand M.A., Song J., Aarts J., Barr P.J., Berger Z., Cochran N., Frosch D., Galasiński D., Gulbrandsen P. (2017). A Three-Talk Model for Shared Decision Making: Multistage Consultation Process. BMJ.

[10] Department of Health (2010). Equity and Excellece: Liberating the NHS.

[11] Department of Health (2012). Liberating the NHS: No Decision about Me, without Me.

[12] National Collaborating Centre for Mental Health (2021). Shared Decision Making.

[13] Charles C., Gafni A., Whelan T. (1997). Shared Decision-Making in the Medical Encounter: What Does It Mean? (Or It Takes at Least Two to Tango). Soc. Sci. Med..

[14] Alguera-Lara V., Dowsey M.M., Ride J., Kinder S., Castle D. (2017). Shared Decision Making in Mental Health: The Importance for Current Clinical Practice. Australas. Psychiatry.

[15] Carrotte E.R., Hartup M.E., Lee-Bates B., Blanchard M. (2021). “I Think That Everybody Should Be Involved”: What Informs Experiences of Shared Decision-Making in Supporting People Living with Schizophrenia Spectrum Disorders?. Patient Educ. Couns..

[16] Hamann J., Neuner B., Kasper J., Vodermaier A., Loh A., Deinzer A., Heesen C., Kissling W., Busch R., Schmieder R. (2007). Participation Preferences of Patients with Acute and Chronic Conditions. Health Expect..

[17] Hamann J., Mendel R.T., Fink B., Pfeiffer H., Cohen R., Kissling W. (2008). Patients’ and Psychiatrists’ Perceptions of Clinical Decisions During Schizophrenia Treatment. J. Nerv. Ment. Dis..

[18] Hamann J., Mendel R., Schebitz M., Reiter S., Bühner M., Cohen R., Berthele A., Kissling W. (2010). Can Psychiatrists and Neurologists Predict Their Patients’ Participation Preferences?. J. Nerv. Ment. Dis..

[19] Hamann J., Cohen R., Leucht S., Busch R., Kissling W. (2005). Do Patients With Schizophrenia Wish to Be Involved in Decisions About Their Medical Treatment?. Am. J. Psychiatry.

[20] Haugom E.W., Stensrud B., Beston G., Ruud T., Landheim A.S. (2022). Experiences of Shared Decision Making among Patients with Psychotic Disorders in Norway: A Qualitative Study. BMC Psychiatry.

[21] Lorem G.F., Frafjord J.S., Steffensen M., Wang C.E. (2014). Medication and Participation: A Qualitative Study of Patient Experiences with Antipsychotic Drugs. Nurs. Ethics.

[22] Park S.G., Derman M., Dixon L.B., Brown C.H., Klingaman E.A., Fang L.J., Medoff D.R., Kreyenbuhl J. (2014). Factors Associated With Shared Decision–Making Preferences Among Veterans With Serious Mental Illness. Psychiatr. Serv..

[23] De las Cuevas C., Peñate W. (2014). To What Extent Psychiatric Patients Feel Involved in Decision Making about Their Mental Health Care? Relationships with Socio-Demographic, Clinical, and Psychological Variables. Acta Neuropsychiatr..

[24] Drivenes K., Haaland V.Ø., Hauge Y.L., Vederhus J.-K., Irgens A.C., Solli K.K., Regevik H., Falk R.S., Tanum L. (2020). Discrepancy in Ratings of Shared Decision Making Between Patients and Health Professionals: A Cross Sectional Study in Mental Health Care. Front. Psychol..

[25] National Collaborating Centre for Mental Health (2014). Psychosis and Schizophrenia in Adults: Prevention and Management.

[26] García-Herrera Perez Bryan J.M., Hurtado Lara M.M., Quemada Gonzalez C., Nogueras Morillas E.V., Bordallo Aragón A., Martí García C., Millán Carrasco A., Rivas Guerrero F., Morales Asensio J.M., de Salud S.A. (2019). Guía de Práctica Clínica Para El Tratamiento de La Psicosis y La Esquizofrenia [Clinical Guidelines for the Treatment of Psychoses and Schizophrenia].

[27] Pottick K.J., Warner L.A., Stoep A.V., Knight N.M. (2014). Clinical Characteristics and Outpatient Mental Health Service Use of Transition-Age Youth in the USA. J. Behav. Health Serv. Res..

[28] Morrison A.P., Hutton P., Shiers D., Turkington D. (2012). Antipsychotics: Is It Time to Introduce Patient Choice?. Br. J. Psychiatry.

[29] Delman J., Clark J.A., Eisen S.V., Parker V.A. (2015). Facilitators and Barriers to the Active Participation of Clients with Serious Mental Illnesses in Medication Decision Making: The Perceptions of Young Adult Clients. J. Behav. Health Serv. Res..

[30] Ashoorian D.M., Davidson R.M. (2021). Shared Decision Making for Psychiatric Medication Management: A Summary of Its Uptake, Barriers and Facilitators. Int. J. Clin. Pharm..

[31] Stovell D., Morrison A.P., Panayiotou M., Hutton P. (2016). Shared Treatment Decision-Making and Empowerment Related Outcomes in Psychosis: Systematic Review and Meta-Analysis. Br. J. Psychiatry.

[32] Thomas E.C., Suarez J., Lucksted A., Siminoff L., Hurford I., Dixon L., O’Connell M., Salzer M. (2022). Treatment Decision Making Needs among Emerging Adults with Early Psychosis. Early Interv. Psychiatry.

[33] Kokanović R., Brophy L., McSherry B., Flore J., Moeller-Saxone K., Herrman H. (2018). Supported Decision-Making from the Perspectives of Mental Health Service Users, Family Members Supporting Them and Mental Health Practitioners. Aust. N. Z. J. Psychiatry.

[34] Shields M., Scully S., Sulman H., Borba C., Trinh N.-H., Singer S. (2019). Consumers’ Suggestions for Improving the Mental Healthcare System: Options, Autonomy, and Respect. Community Ment. Health J..

[35] Barbato A., Bajoni A., Rapisarda F., D’Anza V., De Luca L.F., Inglese C., Iapichino S., Mauriello F., D’Avanzo B. (2014). Quality Assessment of Mental Health Care by People with Severe Mental Disorders: A Participatory Research Project. Community Ment. Health J..

[36] Laugharne R., Priebe S. (2006). Trust, Choice and Power in Mental Health: A Literature Review. Soc. Psychiatry Psychiatr. Epidemiol..

[37] Stromwall L.K., Hurdle D. (2003). Psychiatric Rehabilitation:An Empowerment-Based Approach to Mental Health Services. Health Soc. Work.

[38] Taylor S.J., Bogdan R., DeVault M. (2015). Introduction to Qualitative Research Methods: A Guidebook and Resource.

[39] Guba E., Lincoln Y. (2000). Paradigmatic Controversies, Contradictions, and Emerging Confluences. Handbook of Qualitative Research.

[40] Gleeson J., Larsen T.K., McGorry P. (2003). Psychological Treatment in Pre- and Early Psychosis. J. Am. Acad. Psychoanal. Dyn. Psychiatry.

[41] Bighelli I., Salanti G., Huhn M., Schneider-Thoma J., Krause M., Reitmeir C., Wallis S., Schwermann F., Pitschel-Walz G., Barbui C. (2018). Psychological Interventions to Reduce Positive Symptoms in Schizophrenia: Systematic Review and Network Meta-Analysis. World Psychiatry.

[42] Frawley E., Cowman M., Lepage M., Donohoe G. (2021). Social and Occupational Recovery in Early Psychosis: A Systematic Review and Meta-Analysis of Psychosocial Interventions. Psychol. Med..

[43] Hurtado M.M., Triviño M., Arnedo M., Roldán G., Tudela P. (2016). Are Executive Functions Related to Emotional Intelligence? A Correlational Study in Schizophrenia and Borderline Personality Disorder. Psychiatry Res..

[44] Chan K.K.S., Mak W.W.S. (2012). Shared Decision Making in the Recovery of People with Schizophrenia: The Role of Metacognitive Capacities in Insight and Pragmatic Language Use. Clin. Psychol. Rev..

[45] Légaré F., Thompson-Leduc P. (2014). Twelve Myths about Shared Decision Making. Patient Educ. Couns..

[46] Howe L., Tickle A., Brown I. (2014). ‘Schizophrenia Is a Dirty Word’: Service Users’ Experiences of Receiving a Diagnosis of Schizophrenia. Psychiatr. Bull..

[47] Pitt L., Kilbride M., Welford M., Nothard S., Morrison A.P. (2009). Impact of a Diagnosis of Psychosis User Led Qualitative Study. Psychiatr. Bull..

[48] Baup H., Verdoux H. (2017). Frequency and Pattern of Internet Use in Patients with Schizophrenia or Bipolar Disorders Seeking Medical Information. Psychiatr. Res..

[49] Trefflich F., Kalckreuth S., Mergl R., Rummel-Kluge C. (2015). Psychiatric Patients׳ Internet Use Corresponds to the Internet Use of the General Public. Psychiatry Res..

[50] Wood L., Williams C., Billings J., Johnson S. (2019). The Therapeutic Needs of Psychiatric In-Patients with Psychosis: A Qualitative Exploration of Patient and Staff Perspectives. BJPsych Open.

[51] Tingleff E.B., Bradley S.K., Gildberg F.A., Munksgaard G., Hounsgaard L. (2017). “Treat Me with Respect”. A Systematic Review and Thematic Analysis of Psychiatric Patients’ Reported Perceptions of the Situations Associated with the Process of Coercion. J. Psychiatr. Ment. Health Nurs..

[52] Paksarian D., Mojtabai R., Kotov R., Cullen B., Nugent K.L., Bromet E.J. (2014). Perceived Trauma During Hospitalization and Treatment Participation Among Individuals With Psychotic Disorders. Psychiatr. Serv..

[53] Hurtado Lara M.M., Villena-Jimena A., Quemada Gonzalez C., Morales Asensio J.M. (2021). “I Do Not Know Where It Comes from, I Am Suspicious of Some Childhood Trauma’association of Trauma with Psychosis According to the Experience of Those Affected. Eur. J. Psychotraumatology.

[54] Zinkler M. (2019). Supported Decision Making in the Prevention of Compulsory Interventions in Mental Health Care. Front. Psychiatry.

[55] Goulet M.H., Larue C., Dumais A. (2017). Evaluation of Seclusion and Restraint Reduction Programs in Mental Health: A Systematic Review. Aggress. Violent Behav..

[56] Allerby K., Goulding A., Ali L., Waern M. (2022). Increasing Person-Centeredness in Psychosis Inpatient Care: Staff Experiences from the Person-Centered Psychosis Care (PCPC) Project. BMC Health Serv. Res..

[57] Braun E., Gaillard A.-S., Vollman J., Gather J., Scholten M. (2022). Mental Health Service Users’ Perspectives on Psychiatric Advance Directives: A Systematic Review. Psychiatr. Serv..

[58] Browne J., Nagendra A., Kurtz M., Berry K., Penn D.L. (2019). The Relationship between the Therapeutic Alliance and Client Variables in Individual Treatment for Schizophrenia Spectrum Disorders and Early Psychosis: Narrative Review. Clin. Psychol. Rev..

[59] Browne J., Wright A.C., Berry K., Mueser K.T., Cather C., Penn D.L., Kurtz M.M. (2021). The Alliance-Outcome Relationship in Individual Psychosocial Treatment for Schizophrenia and Early Psychosis: A Meta-Analysis. Schizophr. Res..

[60] McCabe R., Khanom H., Bailey P., Priebe S. (2013). Shared Decision-Making in Ongoing Outpatient Psychiatric Treatment. Patient Educ. Couns..

[61] Hurtado M.M., Villena A., Vega A., Amor G., Gomez C., Morales-Asencio J.M. (2020). ‘I have anxiety, but I have values and preferences’ Experiences of users with generalized anxiety disorder: A qualitative study. Int. J. Ment. Health Nurs..

