# Supplementary material for: “It’s That They Treated Me Like an Object”: A Qualitative Study on the Participation of People Diagnosed with Psychotic Disorders in Their Health Care
Source: Int J Environ Res Public Health. 2023 Mar 5;20(5):4614. doi: 10.3390/ijerph20054614 (PMC10002244; doi:10.3390/ijerph20054614)
Supplement: Supplementary file 1 [file ijerph-20-04614-s001.zip › ijerph-2166630-supplementary.pdf]

Interview Script

Good afternoon and welcome everybody. We have invited you to this meeting because we would like to know your experience regarding participation in the care you have received from the health system since you arrived until now. First of all, we would like to explain what we mean by "participation". We understand participation as the extent to which you have collaborated when making decisions regarding your treatment, the pharmacological treatment, the therapies that have been offered, if you have felt that you have been asked for your opinion, if it was easy to communicate with the professionals, etc. So, if you think so, we are going to ask you some questions about this topic. Any questions?

FIRST CONTACT

How and when was the first contact with the health services? How did they access? What professionals treated you? Did it help someone? Description.

Did you realize on your own that something was happening to you? Did someone advice you? If someone close to you advise you, how did you feel? Did you think it was a good idea to look for help? Did you oppose...?

Describe your first consultation for this health problem. Were you alone or accompanied? Did professional explain anything to you about what was happening to you? Did they prescribe something to you? What information did they give you? How did you feel? Did you feel at ease telling your problem?

SHARE INFORMATION

What elements helped you feel confident to talk about what was happening to you (at the time or later)? Who gave you that confidence?

What did they tell you about what was happening to you? What information did professional give to you? Did you look elsewhere for information? Did you share any doubts with the professional?

OFFERED TREATMENTS

What was the first therapeutic option they offered you? When did they offer you other options?

Have you left the medication on your own at any time? What made you not share it with professionals? Why didn't you share this decision with your professional?

PARTICIPATION MODES

Was your participation different depending on the device in which you were being attended (community mental health unit, therapeutic community, hospitalization…)?

If you have had major physical illnesses, have you noticed if you were more involved in making decisions about your treatment than with the mental disorder?

What do you think about having family members accompany patients when they are admitted or during consultations in the community mental health unit?

Did you miss that the professionals took your point of view more into account?

What obstacles of yours did you observe when participating in your treatment decision process? And of the others?

ADVANCE DIRECTIVES

Do you know what advance wills are in mental health? Has any professional told you something about it, has encouraged you to do it?

REIFICATION

Have you ever felt treated as if you were a thing, a person who was not capable, as if you did not have a voice or a vote? Describe the situations. What has made you feel this way?
